# Vaccination in secondary school students expedites rubella control and prevents congenital rubella syndrome

**DOI:** 10.1186/s12879-016-2046-5

**Published:** 2016-11-30

**Authors:** Hanqing He, Rui Yan, Xuewen Tang, Yang Zhou, Xuan Deng, Shuyun Xie

**Affiliations:** Zhejiang Provincial Center for Disease Control and Prevention, Hangzhou, 310051 People’s Republic of China

**Keywords:** Rubella, Immunity, Rubella containing vaccine (RCV), Female

## Abstract

**Background:**

In order to control the spread of rubella and reduce the risk for congenital rubella syndrome, an additional rubella vaccination program was set up for all secondary school students since 2008 in Zhejiang, China.

**Methods:**

We conducted a descriptive analysis of rubella incidence among different age groups from 2005 to 2015 and a serosurvey of female subjects aged 15–39 years to understand the possible effects of this immunization program.

**Results:**

The average annual rubella incidence rate had decreased from 15.86 per 100,000 population (2005–2007) to 0.75 per 100,000 population (2013–2015) in Zhejiang. The decrease in the rate of rubella incidence in girls aged 15–19 years was more accelerated (from 138.30 to 0.34 per 100,000) than in the total population during 2008–2015 (from 32.20 to 0.46 per 100,000). Of 1225 female subjects in the serosurvey, 256 (20.9%) were not immune to rubella. The proportion of subjects immune to rubella was significantly different among different age groups (Wald *χ*2 = 22.19, *p* = 0.000), and subjects aged 15–19 years old had the highest immunity (88.0%). Rubella antibody levels were significantly lower in women aged 25–30 years with 26.7% of them not immune, followed by the group aged 20–24 years (25.0%) and 30–35 years (24.5%).

**Conclusions:**

Rubella vaccine included in the Expanded Program on Immunization together with vaccination activities for secondary school students can help in rubella control, particularly in targeted age groups in the program. Seroprevalence of antibodies to the rubella virus amongst the female population within childbearing age in Zhejiang, China, is still too low to provide immunity. In addition to vaccination programs in the secondary schools, rubella vaccination should also be encouraged in women of childbearing age, which can be done effectively combined with pre-marital examination in China.

## Background

Rubella, primarily a childhood disease, is generally benign and asymptomatic [1]. It is reported that up to a half of rubella infection may be subclinical or unapparent [2]. Rubella can result in some detrimental results including miscarriage, fetal death, and congenital rubella syndrome (CRS) when infection with the virus occurs in early pregnancy, particularly during the first 16 weeks among non-immunized women [3, 4]. Fortunately, rubella is a vaccine-preventable disease, and the rubella-containing vaccine (RCV) has demonstrated high safety and good effectiveness. Considering the heavy burden of CRS, RCV is recommended in the national children immunization schedules by the World Health Organization [5].

**Table 1:** Demographic characteristics of paricipants included in the serosurvey, Zhejiang, 2015

Age (years)	Changxin		Liandu		Total	
	N	Percent(%)	N	Percent(%)	N	Percent(%)
15y-	115	19.20	135	21.57	250	20.41
20y-	139	23.21	117	18.69	256	20.90
25y-	126	21.04	129	20.61	255	20.82
30y-	116	19.37	122	19.49	238	19.43
35-39y	103	17.20	123	19.65	226	18.45
Total	599	100	626	100	1225	100

**Table 2:** Odds Ratios for Non immunity to Rubella between age groups in female population

Age groups	Seropositive rate (%)	*p*	*OR* (95%CI)
15y-	88.00	-	-
20y-	75.00	0.000	2.44 (1.52–3.93)
25y-	73.33	0.000	2.67 (1.66–4.27)
30y-	76.47	0.001	2.26 (1.39–3.67)
35-39y	83.19	0.135	1.48 (0.88–2.49)

Note:“-”reference groups

Zhejiang province is a developed province in eastern China. RCV was introduced for children in Zhejiang since 1994, but recipients had to pay for the vaccine until 2007. The rubella vaccine coverage in children was less than 50% in Zhejiang as many other places in China from 1994 to 2007 [6, 7]. RCV was included into the Expanded Program on Immunization (EPI) at the end of 2007, and the vaccine was administered free of charge to children at 8 months and 18–24 months of age. The reported coverage rate of rubella was more than 90% in children with mandatory vaccination through the EPI after 2008.

However, the reported incidence rate reached the highest level in 2007 (39.43 per 100,000 population) after rubella was included in the diseases surveillance system in China in 2004 [8]. Many studies have shown that the children vaccination program with RCV may cause the disease to spread to susceptible adults [9, 10]. Considering the increasing risk of CRS, the government of the Zhejiang province set up an additional vaccination campaign for Measles and Rubella Combined Attenuated Live Vaccine (MR) covering all secondary school students irrespective of immunization status since 2008 [11]. This program has shown significant effects toward measles elimination in the Zhejiang province [12], but to what extent it can contribute to rubella control and CRS prevention are unclear yet. Meanwhile, understanding the level of immunity towards rubella in women of childbearing age is also critical to evaluate CRS risk and to provide evidence for implementing further efforts. In the present study, we hypothesize that the vaccination program in secondary school students might be helpful for rubella control, especially in those aged 15–19 years, which was the target age group of this program. We concurrently conducted a descriptive analysis of rubella incidence among different age groups and seroprevalence survey in women 15–39 years of age to explore the effects of the RCV vaccination program in secondary school students.

## Methods

### Ethics statement

The study was approved by the ethics committee of the Zhejiang Provincial Center for Disease Control and Prevention and was conducted in accordance with the Good Clinical Practice guidelines and Declaration of Helsinki. Written informed consent was obtained from all participants or guardians of children (<18 years old) prior to study entry.

### Sources of rubella surveillance information

We collected the data on the annual rubella incidence and age distribution of cases from the National Notifiable Diseases Reporting System (NNDRS) in Zhejiang from 1 January 2005 to 31 December 2015. Information on immunization strategies were obtained from the routine EPI system from the Zhejiang Provincial Center for Diseases Control and Prevention, China.

### Seroprevalence survey

A multistage design was employed to obtain samples from two counties of the Zhejiang Province from July to September 2015, after geographical and economic statuses were considered. Participants were stratified by age into five groups: 15–19 years, 20–14 years, 25–29 years, 30–35 years, and 35–39 years. Sera were excluded from individuals known to be affected by an acute infection, and at least 50 samples were required from each age group in each county. After ethical approval was obtained, each participant was sent a letter explaining the aims of the project and asking for informed consent. Participants were required to provide a 3 mL sample of the venous blood.

### Laboratory test

Serum samples were stored at −70 °C before test. Serological tests were performed at the Measles & Rubella Laboratory in the Zhejiang Provincial Center for Diseases Control and Prevention, which met the Accreditation Criteria for WHO National Measles & Rubella Laboratories. The commercial enzyme-linked immunosorbent assay (ELISA) kit for Rubella (SERION ELISA classic anti-rubella virus IgG, Institute Virion\Serion SDF.FA) was used to measure the level of specific IgG antibodies against rubella virus in the sera. Cut-off and final results were based on the qualitative criteria outlined by the manufacturer, and standard controls in duplicate and negative controls were used in every plate. Seropositivity was defined as a titer of >20 International Units (IU/mL) according to the manufacturer’s guidance.

### Statistical analysis

Incidence and proportion of rubella cases in target subjects (15–19 years) of the immunization program in secondary school students were analyzed for evaluating the epidemiological effects. Seroprevalence of rubella was calculated for subjects in our serosurvey, with 95% confidence intervals (95% CI). Geometric mean concentrations (GMCs) were calculated using log-transformed titers and were reported as back transformed titers. In order to obtain an unbiased GMC, values below the detection threshold (2 IU/mL) were assigned half of the threshold value (1 IU/mL). A logistic regression model and *Wald χ* ^*2*^ test were used to assess the significance of positive seroprevalence proportion between different groups. Kruskal-Wallis *H* test was used to compare the GMCs among groups.

All statistical analyses were performed using SPSS version 20.0 (Statistical Product and Service Solutions, developed by IBM corporation) and a two-tailed *p* value <0.05 was considered statistically significant.

## Results

### Epidemiological effects on the total population

**Fig. 1:**
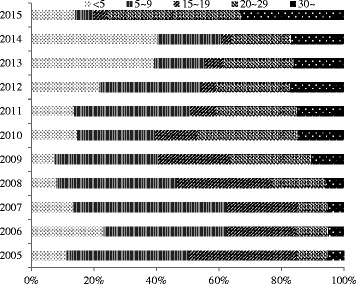
Proportions of Rubella Cases by Age in Zhejiang, 2005–2015

According to NNDRS, the average annual rubella incidence rate had decreased from 15.86 per 100,000 population (2005–2007, before introduction of RCV in EPI) to 0.75 per 100,000 population (2013–2015) in the Zhejiang province. The highest incidence of rubella was 37.87 per 100,000 in 2008, with the highest age specific rate of 160.42 per 100,000 in the 15–19 years age group. After implementing the vaccination campaign of MR in secondary school students at the end of 2008, the proportion of rubella cases in target subjects (15–19 years) decreased from 34.5% in 2005 to 4.3% in 2015. Similarly, the incidence rate of rubella in the 15–19 years age group decreased to 0.35 per 100,000 in 2015, and the rate was lower than that of the total population (0.50 per 100,000). Meanwhile, the ratio of rubella incidences in the 15–19 years age group as compared to that of the total population was also decreased from 4.50 in 2005 to 0.76 in 2015. As we can see from Fig. 1, the largest proportion of rubella cases was in the 20–29 years age group at 42.81% in 2015, while this rate was less than 1% before 2008.

### Epidemiological effects for females

**Fig. 2:**
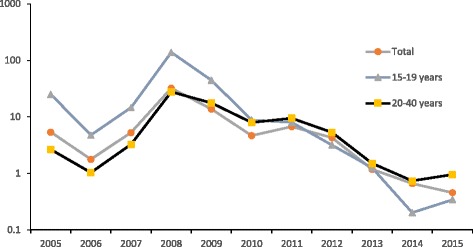
Incidence of Rubella among female population

We can observe in Fig. 2 that the different age specific rubella incidence in women was decreased and follows that of the total population after 2008. The drop rate in the 15–19 age group was faster than in total women, and a lower incidence rate was found in this age group compared with other groups since 2014 (0.34 per 100,000 in the 15–19 years age group vs. 0.46 per 100,000 in the total population, 2015). Conversely, the incidence of rubella in the 20–39 year old women was lower than that of other groups before 2007, and the slow decrease in the incidence rate in this group caused it to later have a higher rate than that of the total population (0.95 per 100,000 in age group 20–39 years, 2015).

#### Immunity effects in females

**Fig. 3:**
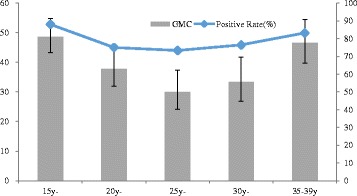
The age-specific seropositivity rate and GMC against rubella

A total of 1225 female subjects of 15 to 39 years old were enrolled in this study (Table 1). The overall seropositivity rate for rubella was 79.1% (95% CI: 76.8–81.4%), with a GMC of 38.5 IU/mL (95% CI: 35.5–41.7 IU/mL). Fig. 3 shows the seropositivity rates and GMC levels of rubella IgG antibodies by age group. The proportion of subjects immune to rubella differed significantly among different age groups (*Wald χ* ^*2*^ = 22.19, *p* = 0.000). For all participants, a U-curve distribution of seropositive rates was found among different age groups.

Rubella antibody levels were significantly lower in women aged 25–30 years with 26.7% of them not immune, followed by those in age groups 20–24 years (25.0%) and 30–35 years (24.5%). Subjects aged 15–19 years old had the highest immunity (88.0%) against rubella, and the seropositive rate was decreased with age until 30 years. The GMC among age groups generally followed the trend of seropositivity and declined obviously from the highest level of 48.6 IU/mL at 15–19 years of age to a lower level of 30.0 IU/mL at 25–29 years of age. There was no significant difference in GMCs among different age groups (*χ* ^*2*^ = 3.77, *p* = 0.439).

As we can see from Table 2, the lowest immunity to rubella was found in the 25–29 years age group, with a seropositivity rate of 73.3%. Women who were 25–29 years old had an odds ratio (OR) of 2.67 (95% CI: 1.66–4.27) for non-immunity to rubella, compared with those in the 15–19 years age group. Both 20–24 years group and 30–34 years age group had seropositivity rates lower than 80%, with OR of 2.44 (95% CI: 1.52–3.93) and 2.26 (95% CI: 1.39–3.67), respectively, for non-immunity to rubella as compared with the 15–19 years age group.

## Discussion

Rubella remains endemic in China and is an important cause of severe birth defects through CRS [13, 14]. RCV was not included in the Chinese EPI until 2007. Low coverage rate of RCV in children persisted since its introduction in Zhejiang in 1994. Even after including it in the national EPI, RCV was only recommended for children of ages 0–14 years. High incidence and increasing proportion of rubella cases in adults in 2008 was a warning that introducing a RCV program in childhood may paradoxically increase the proportion of girls reaching puberty who are susceptible to rubella [15]. An additional RCV program covering all secondary school students was set up in Zhejiang since 2008, to expedite rubella control and reduce the risk of CRS. We performed a descriptive analysis of rubella cases from 2005 to 2015 and a seroprevalence survey of women aged 15–39 years to understand the effects of this program, especially in those target subjects aged 15–19 years.

Our study showed that both the incidence and proportion of rubella cases in target subjects (15–19 years) decreased after implementing the immunization campaign of MR in secondary school students since 2008. Meanwhile, the ratio of rubella incidences in the 15–19 years age group also decreased from 4.50 in 2005 to 0.76 in 2015 as compared to that of the total population. In addition, we found that subjects aged 15–19 years had the highest immunity (88.0%) and GMC antibody level against rubella. This was mainly caused by the vaccination program with the MR vaccine in secondary school students, similar to its effect on the improvement of measles immunity [11]. Currently, most students in secondary schools do not have an immunization history with RCV; thus, the vaccination program can improve the rubella antibody level for protection in these age groups [16]. The rapidly decreasing incidence and higher immunity for rubella in the 15–19 years age group indicated that the vaccination program in secondary school students may expedite rubella control in target subjects.

In consideration of the high disease burden of CRS, rubella prevention should be implemented among adolescent girls and women of childbearing age. Some countries set up the rubella vaccination program only for young women; however, this strategy had proved to be ineffective for preventing the spread of rubella. A supplementary vaccination for male adults was recommended to achieve rubella elimination after an outbreak in Japan [17]. Therefore, we called for a vaccination program in secondary schools covering both male and female students [18]. It is more convenient to conduct an immunization program in students when they are still under the mandatory education system of China. In addition, combination measles-rubella vaccines should be encouraged in the vaccination programs. The most important goal of public health is the elimination of measles together with rubella. The measles elimination program is already one of the most prioritized infectious diseases elimination programs in China; rubella and CRS prevention programs may benefit from the established measles control campaigns [19]. Previous studies showed that adults are becoming an important target of measles in China [11, 20], highlighting that the combined vaccine should be considered as a practical policy option. Therefore, a vaccination program with a combination vaccine may be the optimal choice to enhance immunity both for measles and for rubella [11].

Despite the decrease in the incidence of rubella after RCV was included in the EPI (<1 per 100,000 since 2014) in Zhejiang, this study demonstrated that more than one in five women aged 15–39 years were not immune to rubella. This immunity level is not enough for effective control of rubella in a population [21], and the rate is similar to that reported in other eastern provinces of China [19]. A study from Germany showed that the seropositivity for the rubella antibody was estimated at 89.3% and 86.0% among boys and girls, respectively [22]. Low rubella immunity in pregnant women has also been reported in Canada [23].

Our results point toward a potential risk of CRS according to the increasing proportion of rubella cases in women within the conventional childbearing age range of 20–39 years. There were 42.81% cases in the age group of 20–29 years in 2015, while this rate was less than 10% before the 2008. The incidence rate of rubella in 20–39 year old women was higher than that of the total population in 2015. The highest susceptibility to rubella was found in women aged 25–29 years, with almost one-third of them being not immune. The lower immunity to rubella was also observed in women aged 20–24 years and 30–34 years. When compared with the 15–19 years age group, the ORs of those non-immune to rubella was 2.67 (95% CI: 1.66–4.27) in the 25–29 years age group. Most of these subjects were born in an era when rubella vaccine was just introduced in China, and there was lack of naturally acquired immunity for protection during their childhood due to lower prevalence of rubella [7, 9]. Therefore, rubella IgG levels were usually lower in these subjects than in those born before the introduction of the rubella vaccine [24, 25]. Meanwhile, most of them were not vaccinated with the rubella vaccine because of the low coverage rate during that period. Those women aged 20–35 years who were in the most probable period for childbearing in China, had a higher risk of rubella, which required attention for CRS prevention. However, the current immunization program cannot cover these age groups, and the long-term effects of the vaccination campaign in secondary school students are still unclear. The lower immunity to rubella among 20–35-year-old women also indicates that a targeted vaccination effort for adults is required, but it will be very difficult to implement a campaign targeting adults in China. In order to reduce the possible risk of CRS, physicians should encourage rubella vaccination in women of childbearing age. The test for antibody levels against the rubella virus was included in the national free pre-marital health checkup for all women of childbearing age in China and free rubella vaccination should also be administered to those women who test negative for the antibody to rubella during the examination.

Our study has some Limitations. This study has a cross-sectional design and the method involves only an indirect measure of trend. Additionally, the unavailability of reliable vaccination records of adults restricted the options for a more detailed analysis to investigate the associations on immunity. Further studies are needed to track the seroprevalence for rubella and to assess the long-term protective efficacy of vaccination programs in secondary school students.

## Conclusions

Our findings indicated that vaccination programs using RCV in secondary school students can curb the spread of rubella and may reduce the risk of CRS. However, seroprevalence of antibodies to rubella amongst the female population within child bearing age in Zhejiang, China, is still too low to provide immunity. We strongly suggest that in addition to implementing vaccination programs in secondary schools, rubella vaccination should also be encouraged in women of childbearing age, which can be carried out effectively when combined with the pre-marital examination in China.

## Abbreviations

CRS: Congenital rubella syndrome
EPI: Expanded Program on Immunization
MR: Measles and Rubella Combined Attenuated Live Vaccine
NNDRS: National Notifiable Diseases Reporting System
RCV: Rubella containing vaccine
